# Colorectal cancer in a 13‐year‐old with constitutional mismatch repair deficiency and *MUTYH* heterozygosity

**DOI:** 10.1002/jpr3.70001

**Published:** 2025-02-19

**Authors:** Chloe J. Cohan, Caroline Chinchilla Putzeys, Brianna Pruniski, Paul Tran

**Affiliations:** ^1^ Creighton University School of Medicine Phoenix Arizona USA; ^2^ Division of Gastroenterology Phoenix Children's Hospital Phoenix Arizona USA; ^3^ Division of Genetics and Metabolism Phoenix Children's Hospital Phoenix Arizona USA

**Keywords:** café‐au‐lait macules, constitutional mismatch repair deficiency (CMMRD), Lynch syndrome, neurofibromatosis type 1 (NF1), pediatric colorectal cancer

## Abstract

Constitutional mismatch repair deficiency (CMMRD) is a rare hereditary cancer syndrome resulting from biallelic mutations in DNA mismatch repair (MMR) genes that lead to early‐onset cancers in children, including lymphoma and colorectal cancer (CRC). This case report presents a 13‐year‐old boy diagnosed with CMMRD due to a homozygous *MSH6* mutation and a heterozygous *MUTYH* mutation. The patient's initial misdiagnosis as neurofibromatosis type 1 (NF1) highlights the overlap between CMMRD and NF1, as their overlapping genetic pathologies can yield similar clinical manifestations. This case emphasizes the complexity of genetic diagnoses, particularly when multiple predispositions like MMR and *MUTYH* mutations coexist. Accurate identification of CMMRD and associated mutations is crucial for timely management and genetic counseling, given its significant implications for cancer risk and treatment strategies.

## 1 INTRODUCTION

Colorectal cancer (CRC) is rare in children, with most diagnoses occurring in individuals over the age of 50 years. Currently recognized CRC predisposition syndromes in younger individuals include constitutional mismatch repair deficiency (CMMRD), Lynch syndrome, and familial adenomatous polyposis (FAP).^1^

CMMRD is a rare childhood hereditary cancer predisposition syndrome characterized by a biallelic mutation in one of four DNA mismatch repair (MMR) genes, *MLH1, MSH2, MSH6*, or *PMS2*. The general prevalence of pathogenic variants of these genes is approximately 0.051% for *MLH1*, 0.035% for *MSH2*, 0.132% for *MSH6*, and 0.140% for *PMS2*.^2^ CMMRD increases the risk for childhood‐onset CRC, gliomas, glioblastomas, non‐Hodgkin lymphoma, and endometrial and urinary tract cancers. In contrast, monoallelic mutations in these same four DNA MMR genes result in Lynch syndrome, associated with adult‐onset colorectal, endometrial, gastric, ovarian, pancreatic, urinary tract, prostate, biliary tract, small intestinal, brain, and sebaceous gland cancers.^3^, ^4^ Mutations in other genes involved in DNA repair can also increase cancer risk. For instance, monoallelic mutations of the *MUTYH* gene confer a slightly elevated lifetime risk for colorectal, duodenal, ovarian, bladder, and endocrine cancers.^5^

CMMRD has been documented in few cases to be linked with the development of various cancers including colon cancer in young adult patients.^6^ Previous literature describes cases of children with parents diagnosed with CMMRD syndrome, particularly those with germline biallelic *MSH6* mutations, presenting with café‐au‐lait macules and a history of multiple family cancers.^7^ However, reported cases of CRC development in children with multiple inherited cancer‐related genes are not well‐represented in current literature. This report describes the case of a boy with CMMRD, a monoallelic *MUTYH* mutation, and biparental history of Lynch syndrome who developed lymphoma and early‐onset CRC.

## 2 CASE REPORT

A male infant initially underwent a genetics consultation in 2011 for suspicion of neurofibromatosis type 1 (NF1) due to cutaneous findings of café‐au‐lait macules. He did not meet the clinical criteria and therefore did not undergo testing for NF1 at that time. In 2012, at 16 months of age, he was first diagnosed with T‐lymphoblastic lymphoma and underwent chemotherapy treatment with nelarabine, with subsequent remission. Shortly after, his mother was diagnosed with endometrial adenocarcinoma and found to have Lynch syndrome and a heterozygous *MUTYH* mutation. His father was also tested and found to have Lynch syndrome.

The patient continued to be managed with a presumptive underlying diagnosis of NF1. At age nine, his T‐lymphoblastic lymphoma recurred, prompting germline testing. A multi‐cancer genetic panel revealed a homozygous *MSH6* pathogenic mutation and a heterozygous *MUTYH* pathogenic mutation. Combined with clinical manifestations like café‐au‐lait macules, these results led to a formal diagnosis of CMMRD, rather than NF1. The patient underwent reinduction chemotherapy and again achieved remission.

**Figure 1:**
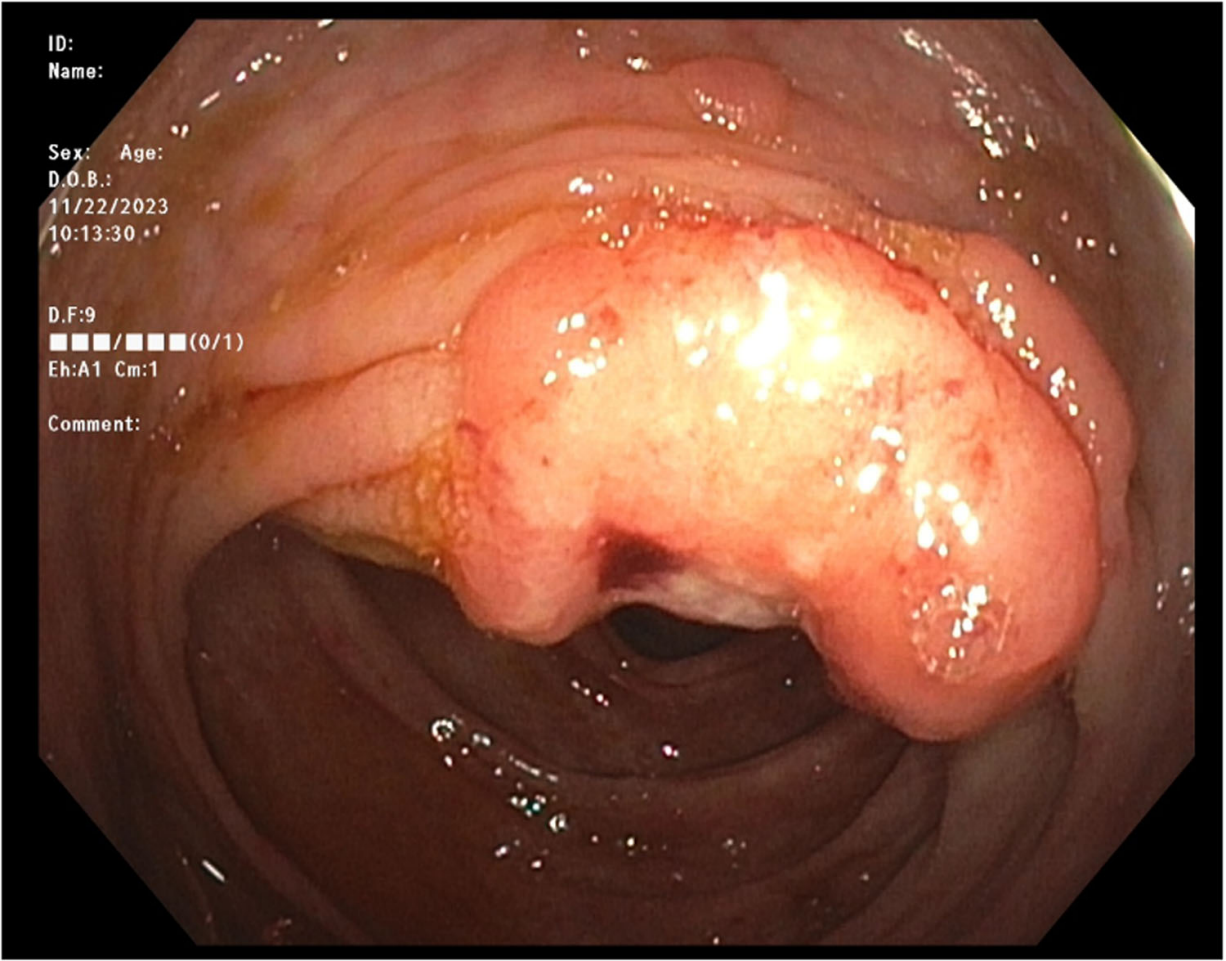
Colonic adenocarcinoma. Large firm sessile mass with superficial ulceration and overlying area of eschar located in ascending colon. Attempted submucosal lifting successful but hot snare removal was unsuccessful.

After referral from hematology‐oncology, the patient began endoscopic surveillance for CRC at 11 years old when he developed intermittent hematochezia. Endoscopic evaluation revealed 50‐100 colonic polyps, with pathology showing tubular adenomas without high‐grade dysplasia. Endoscopic surveillance proceeded over time according to guidelines from the US Multi‐Society Task Force on Colorectal Cancer for BMMRD‐related CRC screening. A repeat procedure 6 months later showed increased polyp burden but was deemed manageable with endoscopic surveillance. Six months afterwards, he presented with significant weight loss and recurrent hematochezia. Endoscopic evaluation showed increased polyposis and a large, immobile, ulcerated right colonic mass that could not be removed endoscopically (Figure 1). While an abdominal computed tomography reassured against other masses, he underwent a total proctocolectomy with ileal‐pouch anal anastomosis. Surgical pathology revealed a well‐differentiated colonic adenocarcinoma. The patient is currently doing well postoperatively.

## 3 DISCUSSION

This case highlights the clinical overlap between CMMRD and NF1, as both can involve mutations to the *NF1* gene. CMMRD patients may develop somatic *NF1* mutations due to defective mismatch repair, while classical NF1 involves germline mutations affecting all cells. This genetic overlap can lead to shared features such as café‐au‐lait macules and Lisch nodules, sometimes causing CMMRD to be misdiagnosed as NF1. Thus, in patients presenting with café‐au‐lait macules, CMMRD should remain on the differential, especially in patients with familial history of cancer syndromes.

The gold standard for a definitive diagnosis of CMMRD requires genetic testing, typically through a blood sample, to identify a germline biallelic mutation in one of the four MMR genes. This is combined with clinical features such as early‐onset cancers (e.g. brain tumors, hematological malignancies, and CRCs) often in young individuals, as well as nonmalignant signs like café‐au‐lait spots or a family history of similar cancers.^8^ Given the patient's biparental history of Lynch syndrome and the mother's diagnosis of endometrial adenocarcinoma, the risk of inheriting two mutated DNA mismatch repair alleles—and developing CMMRD—should have been considered earlier. Despite the eventual diagnosis of CMMRD, the patient was not referred to gastroenterology until hematochezia occurred. A higher index of suspicion and broader criteria for early genetic testing and surveillance in patients presenting with café‐au‐lait macules, coupled with a personal history of CMMRD‐related malignancies, could have led to timely intervention before cancer signs emerged. While genetic testing is not routinely performed for patients with café‐au‐lait macules alone, their presence, alongside a history of lymphoma and parental Lynch syndrome diagnoses, represented a missed opportunity for earlier diagnosis and intervention before the patient's first colon cancer. This case underscores the importance of effectively communicating genetic test results with care teams to ensure timely cancer surveillance in predisposition syndromes. Additionally, sharing a diagnosis with family members allows relatives to be assessed for related syndromes and monitored appropriately. In cases of CMMRD, siblings should undergo genetic testing and ongoing evaluation for features of the condition.

Although CRCs associated with CMMRD are diagnosed at an average age of 16 years, it is important to recognize that they may occur earlier; cases requiring more aggressive intervention at an early stage must not be overlooked due to low suspicion.^9^ Although a colonoscopy was performed 6 months before diagnosis of CRC, a higher index of suspicion for potential malignancy might have allowed for complete polypectomy before progression to adenocarcinoma. In this case, while the largest polyps found were removed at each endoscopy, there was potentially room for removal of more polyps that could have fit the criteria. This certainly raises the question of whether a more extensive polypectomy could have been performed and served beneficial. Due to the low incidence of CRC in children, pediatric endoscopic training is not highly focused on cancer surveillance measures. This case emphasizes that training should include heightened awareness of considering rare hereditary colon cancer syndromes in differential diagnoses as well as in determining aggressiveness of treatment. Training should also emphasize the importance of thoroughly and individually assessing each polyp for fitting the criteria for polypectomy, as overlooking even one could have drastic consequences. Lower thresholds for complete polypectomies should be considered in cases of aggressive CRC predisposition syndromes such as CMMRD.

From a genetic counseling perspective, this case illustrates how broader testing can complicate risk analysis due to additional findings, such as an *MUTYH* mutation. Given the rarity of CMMRD, limited data exists on how multiple predispositions interact and whether the risks are independent, additive, or compounding. In this case, an additive effect seems plausible, given the younger‐than‐average onset of CMMRD‐related CRC and rapid progression from tubular adenoma to adenocarcinoma within 6 months. This could be due to an expedited adenoma‐to‐carcinoma sequence and/or microsatellite instability linked to CMMRD.^10^ Nonetheless, with few documented cases, the exact effects of concurrent MMR and *MUTYH* mutations on clinical outcomes remain unclear.

Finally, the repeated mention of polyposis syndrome and FAP in the medical record highlights the need for increased awareness of CMMRD in clinical practice. It is crucial to differentiate CMMRD from other syndromes with overlapping features, such as FAP, juvenile polyposis, Turcot syndrome, Li‐Fraumeni syndrome, and Peutz‐Jeghers syndrome, to ensure proper diagnosis and management.^11^ Emphasizing CMMRD in the differential diagnosis of polyposis syndromes could promote earlier detection and more personalized management, improving outcomes.

In summary, this case underscores the importance of a broader approach to genetic testing in individuals presenting with signs consistent with CMMRD, given the significant overlap with other genetic conditions. Additionally, recognizing the potential for CRC in children with two parents with Lynch syndrome can promote earlier detection and intervention and better outcomes. Understanding additional mutations in patients with CMMRD is crucial for optimizing diagnosis and management.

## CONFLICT OF INTEREST STATEMENT

The authors declare no conflicts of interest.

## ETHICS STATEMENT

Verbal informed consent was obtained from the parent of the minor patient for the publication of this case report.
